# The Treatment of Pruritus Ani

**Published:** 1904-12-17

**Authors:** 


### THE TREATMENT OF PRURITUS ANI.

Malcolm Morris [1] considers pruritus ani, though often a symptom, to be sometimes an essential disease. It may depend on constitutional causes such as gout, disturbances of the digestive system, albuminuria, and diabetes; in some instances it is a by-product of rheumatism; in others it is induced by some element in the diet, or by the use of tobacco. Among local causes are to be considered hæmorrhoids, fissure, fistula, ulceration, growths, foreign bodies, parasites such as intestinal worms and pediculi, discharges from the bowel, and local cutaneous diseases. Pruritus ani in another group of cases may be a reflex result of anything which obstructs the venous circulation in the lower abdomen, and here disease of the bladder, prostate, uterus, and rectum may be mentioned. As an essential disease the condition must be regarded as a sensory neurosis often originating in emotional disturbances of a depressing character or in a sense of failure or unfitness for the duties and struggles of life. The pruritus is often of great intensity and is frequently paroxysmal, tending to occur when the patient is warm in bed. The scratching it provokes may produce excoriation of the skin and mucous membrane, and secondary lesions—pustular, ulcerative, or eczematous—may follow. The first factor in treatment is to discover and, where possible, to remove the cause, and thus each case needs individual study. Attention to the action of the bowels and the prescription of a bland but nutritious diet are needed in all cases. Similarly it is imperative to prohibit the use of condiments, highly-seasoned dishes, game, and alcohol; coffee also should be forbidden. To prevent auto-intoxication the alimentary canal should be kept free as far as possible from harmful toxins. Measures calculated to effect this are small doses of calomel at bedtime with a saline purgative in the morning, ichthyol (5 grs.) night and morning, or salol. Afterwards the bowels may be regulated by cascara or other laxative; aloes is contra-indicated because of its irritating action on the rectum. Other general measures are tonics, as quinine, arsenic, and nux vomica; the thorough flushing of the system with a weak alkaline water, combined, if necessary, with a residence at a suitable health resort, and in some cases sedatives. In reference to the latter, they should only be used if the indication is a clear one. Opium in particular is to be avoided, as not infrequently it excites itching. If sleep is seriously invaded, succus conii in one-drachm doses thrice daily, or potassium bromide, or chloral hydrate may be cautiously allowed. The first essentials in local treatment are the removal of any localised cause of irritation and the practice of absolute cleanliness. For the latter the use of paper in the ordinary fashion is quite insufficient. To order the use of soap and water after every action of the bowels is a counsel of perfection. But a pledget of cotton-wool dipped in the clean water obtained by refilling the pan of the closet is quite a practicable method. In addition, washing of the anal region night and morning should be practised, and coal-tar soap may be advised for this purpose. Sitz-baths of very hot water are also useful, but some persons get greater relief from cold water.

Local soothing applications are legion, and experience alone will tell which is the most appropriate to an individual case. Cocaine is one of the most valuable. It may be prescribed as a suppository (½ grain in each) or as a 4 per cent. ointment with lanovaseline or boric-acid ointment as a basis. The risk of the cocaine habit must be borne in mind. Menthol may be combined with cocaine or may be ordered as a lotion—10 grains to an ounce of dilute alcohol; its immediate cooling effect is apt to be followed by heat and tingling. Another excellent sedative is a strong solution of bicarbonate of sodium applied in a poultice. Carbolic acid (2 to 6 grains to an ounce of water, or 1 in 20 of olive oil) may be used as a lotion or applied on a compress; it is often highly successful. Of tarry preparations the best are the lotio picis carbonis, 2 drachms in 8 oz. of calamine lotion; tar ointment, a drachm with 20 grains of bismuth subnitrate to an ounce of lard; and oil of cade applied on a compress. To prevent the common nocturnal exacerbation a suppository of belladonna extract (half-grain) is useful. Among antiseptic applications mercurials take the first place. The oleate, calomel ointment, ammoniated mercury ointment, and black wash are all of value; another useful application is formed by dissolving two grains of corrosive sublimate in half an ounce of glycerine and eight ounces of water. Dr. S. D. Johns recommends the application of about 20 grains of calomel after each action of the bowels, the part being first washed and carefully dried; he supplements this local measure by the administration of small doses of magnesium sulphate. Silver nitrate, cauterisation, electricity in various forms are other measures which sometimes prove useful. In purely neurotic cases the less local treatment the better. These patients need tact and firmness; the claim is rather for moral than medicinal tonics. In some instances Dr. Milne Bramwell has succeeded in effecting a cure by means of hypnotic suggestion.

In a characteristic lecture the late Sir W. Mitchell Banks, referring to the subject of pruritus ani, said he had found most good from ordering the patient to sit in very hot water before getting into bed, and then dabbing the affected parts with a solution of carbolic acid as strong as could be borne; in some instances solution of potassium cyanide or of silver nitrate had proved useful. Greasy applications had not in his experience been a success, and there is a certain proportion of the cases in which neither drugs nor special varieties of diet have any effect. Further, in these "idiopathic" cases the itching may be very extreme, and the irritation and loss of sleep may seriously affect both the bodily and mental condition of the patient. In such examples the actual cautery affords some prospect of cure. To apply it the patient should be anæsthetised and placed in the lithotomy position. Any small skin piles should be clipped away with the scissors. If there are not many rugæ, the large bulbous-headed cautery should be used, and the skin for an inch and a-half all round the anal orifice should be "well frizzled." If there are rugæ the small cautery point should be used. It should be introduced into the furrows between the rugæ as well as applied over the top of them. It is only in severe and intractable cases in which no cause for the condition can be found that the cautery should be used, but in these it is "unfailingly curative."

[1] Brit. Med. Jour. Oct. 15, 1904.

